# Interfacial Effect-Based Quantification of Droplet Isothermal Nucleic Acid Amplification for Bacterial Infection

**DOI:** 10.1038/s41598-019-46028-8

**Published:** 2019-07-03

**Authors:** Tiffany-Heather Ulep, Alexander S. Day, Katelyn Sosnowski, Alexa Shumaker, Jeong-Yeol Yoon

**Affiliations:** Department of Biomedical Engineering, The University of Arizona, Tucson, Arizona 85721 United States

**Keywords:** Chemical engineering, Biomedical engineering

## Abstract

Bacterial infection is a widespread problem in humans that can potentially lead to hospitalization and morbidity. The largest obstacle for physicians/clinicians is the time delay in accurately identifying infectious bacteria, especially their sub-species, in order to adequately treat and diagnose such infected patients. Loop-mediated amplification (LAMP) is a nucleic acid amplification method that has been widely used in diagnostic applications due to its simplicity of constant temperature, use of up to 4 to 6 primers (rendering it highly specific), and capability of amplifying low copies of target sequences. Use of interfacial effect-based monitoring is expected to dramatically shorten the time-to-results of nucleic acid amplification techniques. In this work, we developed a LAMP-based point-of-care platform for detection of bacterial infection, utilizing smartphone measurement of contact angle from oil-immersed droplet LAMP reactions. Whole bacteria (*Escherichia coli* O157:H7) were assayed in buffer as well as 5% diluted human whole blood. Monitoring of droplet LAMP reactions was demonstrated in a three-compartment, isothermal proportional-integrated-derived (PID)-controlled chip. Smartphone-captured images of droplet LAMP reactions, and their contact angles, were evaluated. Contact angle decreased substantially upon target amplification in both buffer and whole blood samples. In comparison, no-target control (NTC) droplets remained stable throughout the 30 min isothermal reactions. These results were explained by the pre-adsorption of plasma proteins to an oil-water interface (lowering contact angle), followed by time-dependent amplicon formation and their preferential adsorption to the plasma protein-occupied oil-water interface. Time-to-results was as fast as 5 min, allowing physicians to quickly make their decision for infected patients. The developed assay demonstrated quantification of bacteria concentration, with a limit-of-detection at 10^2^ CFU/μL for buffer samples, and binary target or no-target identification with a limit-of-detection at 10 CFU/μL for 5% diluted whole blood samples.

## Introduction

Bacterial infection is a widespread and common problem that can lead to various complications in humans. Specifically, the existence of antibiotic resistant bacteria^1^ can lead to additional complications, including hospitalization and mortality. These bacteria include *Staphylococcus aureus* and *Escherichia coli* (specifically Shiga-toxin producing type such as O157:H7)^2^. Diagnosis of such bacterial infection have traditionally been made based on the patient’s symptoms, followed by broad-spectrum antibiotic treatments^3^, which have shown positive results in decreasing mortality rates. However, efforts to conduct such diagnoses in a specific and sensitive manner are in high demand. Most importantly, there exist a strong need for identifying antibiotic and drug resistant microbes, monitoring the spread of epidemic to pandemic infectious diseases, and addressing sociopolitical infrastructure disparities^4^.

The largest obstacle for physician/clinicians is the time delay in accurately identifying infectious pathogenic bacteria (especially their sub-species) in order to adequately treat the infected patients. Early detection and subsequent adequate treatment are highly correlated to decreased mortality and hospitalization^5–7^. The current gold standard is bacterial identification through culture-enrichment processes from patient’s specimens (urine, nasal swab, throat swab, blood, serum, tissue biopsy, etc.). Typically, laboratories are equipped with automated culturing systems, where growth curves in enriched media are monitored via carbon dioxide levels, fluorescence, or colorimetric photometry. The greatest disadvantage to such conventional methods of bacterial identification is their inherently long processing time of about 12 to 72 h. Such delays to pathogen identification have been a key obstacle in the early diagnosis and administration of treatment for infected patients. Other pitfalls to bacterial cultures for pathogen identification are the requirement of specialized equipment, personnel, and facilities^5,8^.

Recent attempts to decrease time-to-results for bacterial identification include the integration of molecular diagnostic techniques such as hybridization [i.e. fluorescent *in situ* hybridization (FISH)], nucleic acid amplification [i.e. broad range and multiplexed polymerase chain reaction (PCR)], mass spectroscopy [i.e. matrix-assisted laser desorption/ionization time-of-flight mass spectrometry (MALDI-TOF MS)], and protein analysis^9^. By using such molecular techniques, the time-to-results is expedited since blood samples can be directly analyzed, bypassing the culture-enrichment process. PCR techniques have been more broadly applied in blood borne pathogen detection. However, PCR has been notoriously known for its susceptibility to false negatives due to the overwhelming background human DNA present in whole blood samples. Other components in the specimen such as ions and proteins can also impede the amplification mechanism^8,10^. More importantly, PCR requires three set temperatures (denaturation, annealing, and extension) that are alternated for ~30 cycles. This necessitates a thermal cycler, which is not appropriate for point-of-care (POC) diagnostics.

Isothermal nucleic acid amplification technologies have emerged as an alternative to PCR techniques, where the amplicons are exponentially produced at a constant temperature^11^. Isothermal techniques have been popularly applied in POC systems due to the requirement of only one temperature and the rapid amplification results (as fast as 20 min), making them attractive for use in field and clinical settings. A specific isothermal method that has gained much reprise, and is even available in commercial kits for identification of pathogens, is loop-mediated isothermal amplification (LAMP). The reaction utilizes 4 to 6 primers, rendering it highly specific and capable of amplifying low initial copies of target sequence in a sample. LAMP amplicons are produced by cycling strand displacements and are dumbbell or cauliflower shaped^12,13^.

In the past decade, 146 total publications have been identified using LAMP as a molecular diagnostic technique in POC platforms (Web of Science, using the keywords “loop-mediated amplification,” “diagnosis,” and “point-of-care”). Various detection methods were used with these POC LAMP platforms for confirmation and monitoring of target amplification, including electrochemical^14,15^, fluorescent^16,17^, colorimetric^18–20^, and turbidimetric (due to magnesium pyrophosphate precipitation)^21,22^ detection. While these detection methods have been used for demonstrating LAMP in POC platforms, they are susceptible to many different experimental and/or environmental conditions, such as ambient lighting, contaminants in samples, etc. They also require rather complicated equipment, such as spectrophotometers, optical filters, diffraction gratings, and/or noise filtration circuits.

An alternative detection method is interfacial tension measurement, where droplet height or shape is measured as an indicator of amplification. In fact, interfacial tension has been measured occasionally for droplet or digital PCR (dPCR), to assure droplets (microreactors) are reproducibly formed and maintained with consistent shape and surface energy, especially for high-throughput screening procedures^23–25^. Similarly, due to increased interest in POC applications, digital LAMP (dLAMP) platforms have been developed^26–28^. However, none of these studies have actively utilized interfacial tension as a sensing mechanism for the amplification of target genes^29,30^. It is possible to relate this interfacial tension to molecular self-assembly at the interface (e.g. water-oil) and protein adsorption/kinetics towards the interface^31–35^, and eventually to the quantification of amplified products.

In this work, a LAMP-based POC platform is proposed for identifying bacterial infection. It monitors the changes in interfacial tension, as represented by change in contact angle, of an oil-immersed droplet that contains LAMP reaction mixture, utilizing smartphone-based image processing. Whole bacteria (*Escherichia coli* O157:H7 with *Staphylococcus aureus* as cross-reference) are assayed. Experiments were repeated with diluted human whole blood as a model for human specimen. Specifically, target genes associated with antibiotic resistance are amplified: *rfbE* gene in *E. coli* and *spA* gene in *S. aureus*. Contact angle measurements are conducted for the LAMP droplets with and without target presence sitting on a proportional-integral-derivative (PID)-controlled heater within a disposable polydimethylsiloxane (PDMS)-based platform. An assay time of <15 min or as fast as 5–10 min is desired, in order to not delay the physician’s decision time in infected patients. Low limit of detection (LOD) is also preferred, e.g., as low as 10 CFU/μL sample.

## Methods

### Bacterial samples

*Escherichia coli* O157:H7 (part #0801622; ZeptoMetrix  Corp., Buffalo, NY, USA) and methicillin-sensitive *Staphylococcus aureus* (MSSA; part #0801675; ZeptoMetrix Corp.) samples were cultured in lysogeny broth (LB) Miller’s formula (Molecular Biological International Inc., Irvine, CA, USA) at 37 °C for 8 hours. 1 mL of final concentrated bacterial stocks (10^5^ CFU/µL) were centrifuged at 5.6 *g* for 10 min and resuspended in 1 mL of nuclease-free water. Stock bacterial samples were then serially diluted from 10^8^ CFU/mL (=10^5^ CFU/μL) stock to 10^2^ CFU/mL (=0.1 CFU/μL) in nuclease-free water.

### LAMP reaction mixture

LAMP primers were purchased from Sigma-Aldrich (St. Louis, MO, USA) following literature^36,37^. 10X target-specific primer sets as shown in Table 1 were formulated to contain 16 µM each of FIP and BIP primers, 8 µM each of Loop-F and Loop-B primers, and 2 µM of F3 and B3 primers. OmniAmp™ RNA and DNA LAMP Kit (Lucigen Corp., Middleton, WI, USA) was used. Final mixtures were comprised of 1X reaction buffer, 8 mM MgSO_4_, 150 mM betaine, 0.5X primers, 3X OmniAmp polymerase, 8 mM dNTPs (25 mM each), 1 µL of target bacteria solution, and 0.01% w/v Span® 80 (S6760; Sigma). LAMP reactions were prepared on ice. Samples were amplified conventionally in a PCR tube with mineral oil (M5904; Sigma) to avoid evaporation from heat produced. Thermocycler (MJ Research, Waltham, MA, USA) was programmed at 69 °C for 30 min, followed by refrigeration at 4 °C. For reactions containing whole blood, 50% v/v of nuclease free water in 1 μL target bacteria solution was replaced with 5% v/v whole blood diluted in Plasma-Lyte A (pH 7.4; Baxter, Deerfield, IL, USA). Human whole blood was purchased from ZenBio, Inc. (Research Triangle Park, NC, USA), which is pathogen-free as reported by the vendor. These samples were stored refrigerated in a VACUETTE® Blood Collection Tube with 9NC coagulation sodium citrate 3.2% w/v (454334; Greiner Bio-One International, Monroe, NC, USA). All experiments were performed in the Biosensors Laboratory at the University of Arizona, which is at biosafety level 2 and chemical safety level 2 and in accordance with relevant guidelines and regulations. Since the sample collection was performed by the vendor and the samples were pathogen-free, approval by the institutional review board was not required.

**Table 1:** Sequences of oligonucleotide primers used.

Target	Primer	Sequence (5′-3′)	Size (bp)
*E. coli* O157:H7 *rfbE* gene	F3	AACAGTCTTGTACAAGTCCA	20
	B3	GGTGCTTTTGATATTTTTCCG	21
	FIP	CTCTCTTTCCTCTGCGGTCCGATGTTTTCACACTTATTGGAT	43
	BIP	TAAGGAATCACCTTGCAGATAAACTAGTACATTGGCATCGTGT	43
	LoopF	CCAGAGTTAAGATTGAT	17
	LoopB	CGAAACAAGGCCAGTTTTTTACC	23
*S. aureus* MSSA *spA* gene (Protein A)	F3	AATGACTCTCAAGCTCCAA	19
	B3	CTTTGTTGAAATTGTTGTCAGC	22
	FIP	GCTCTTCGTTTAAGTTAGGCATGTT-TGCGCAACAAAATAAGTTCA	45
	BIP	AAGTCTTAAAGACGATCCAAGCC-TTCGGTGCTTGAGATTCG	41
	LoopB	AGCACTAACGTTTTAGGTGAAGC	23

### Gel electrophoresis

LAMP products were analyzed using gel electrophoresis. 3% w/v agarose gel (A0169; Sigma) in 1X tris-acetate-EDTA (TAE) buffer (35100131; Quality Biological Inc., Gaithersburg, MD, USA) was prepared and placed at 120 V for 50 min with an electrophoresis power supply (FB200; Fischer Scientific, Inc., Pittsburgh, PA, USA). TrackIt™ 100 bp DNA ladder (10488058; Invitrogen, Waltham, MA, USA) was used as a standard for fragment sizing. Gels were stained with ethidium bromide (E1510; Sigma) and imaged under UV light. Gel images were analyzed using ImageJ software (U.S. National Institutes of Health, Bethesda, MD, USA).

### PDMS chip fabrication

Custom acrylonitrile butadiene styrene (ABS) resin mold was 3D-printed using MakerBot Replicator Z18 (MakerBot Industries, Brooklyn, NY, USA) resulting in a box shape of 0.5 mm × 0.5 mm × 1 mm, to create a chamber for conducting droplet-based LAMP reaction. This mold was then adhered to a two-compartment polystyrene Petri dish (99 mm × 15 mm; AB1471; Flinn Scientific, Batavia, IL, USA), resulting in the chip template. Molds were carefully aligned to be parallel to partition for transparent optical window. Sylgard® 184 Silicone Elastomer Clear (4019862; Dow Corning, Midland, MI, USA) polydimethylsiloxane (PDMS) base and curing agent were combined at a 10:1 ratio and poured into chip template salinized by tridecafluoro-1,1,2,2-tetrahydroocyl trichlorosilane (78560-45-9; Gelest Inc., Morrisville, PA, USA). Cured PDMS replica was separated from the chip template and bonded to a microscope glass slide.

### Contact angle monitoring with smartphone

Prior to loading LAMP droplet reactions, mineral oil and PDMS chip was pre-heated for 10 min in order to assure a constant 69 °C temperature. Temperature ramping profile is shown in Supplementary Fig. S1. Through the transparent optical window of the PDMS platform, real-time detection of LAMP amplification was achieved by monitoring the angle tangential to the droplet with respect to the glass surface, during constant applied heat. Images were captured every 30 s for 30 min using the Lapse It application (Interactive Universe, USA) and a smartphone camera (Samsung Galaxy S8, Suwon, South Korea). All images were loaded to ImageJ software and the contact angle was measured by ellipse-fit using the Contact Angle plugin. The absolute change in contact angle was determined with respect to contact angles measured at 30 min, 15 min, 10 min, and 5 min. This procedure was done in triplicates for *E. coli* O157:H7 target amplification varying concentrations of 10^5^, 10^3^, 10^2^, 10, and 0 (NTC) CFU/μL. An unpaired two-tailed t-test compared the changes in droplet height with vs. without target presence (NTC) and cross-reactive samples. Error bars represent standard error (*n* > 3).

### Design and fabrication of the portable device

The smartphone holder, heated chip stage, and temperature controller housing were designed using SolidWorks software (Dassault Systèmes, SolidWorks Corporation, Waltham, MA, USA) and 3D-printed using a Makerbot Replicator Z18 from ABS material. The heated chip stage consists of a thermoelectric heat pump (TEC1-12706, Hebei Int. Trading Co., Ltd., Shanghai, China) and the PDMS platform bound to the microscope glass slide. The heated chip stage is adjustable to the length of a microscope slide to allow optimum positioning of the droplet to the smartphone camera. The temperature controller housing consists of a proportional-integral-derivative (PID) controller (JLD612DC, LightObject, Sacramento, CA, USA) and a 36-guage type K thermocouple (5TC-TT-K-36-36, Omega Engineering, Norwalk, CT, USA) supported by a thermocouple holder that is adjustable perpendicular to the length of a microscope slide. This allowed positioning of a thermocouple into a reference water droplet. The PID controller is powered by a 3 V DC power supply, and continuously monitors the temperature within the reference water droplet. The reference temperature is used to regulate electrical output to a temperature control circuit^38^ which includes a relay (G5SB, Omron Electronic Components, Kyoto, Japan). When the relay is switched to the “on” position, it allows the thermoelectric heat pump to be powered by a 9 V DC power supply. The PID controller was calibrated and programmed to sustain a constant temperature of 69 °C. All compartments of the PDMS chip were filled with mineral oil and heated to steady state temperature before loading the 10 μL LAMP reaction mixture and reference NTC droplet.

## Results

### Conventional LAMP and gel electrophoresis detection

**Figure 1:**
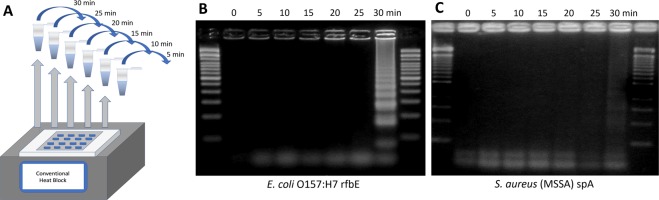
(**A**) Schematic illustration of sequential incubation of 10 μL LAMP reaction mixtures with target *E. coli* O157:H7 (10^5^ CFU/µL) or *S. aureus* (MSSA; 10^5^ CFU/µL) and NTC reactions on conventional heat block for 5, 10, 15, 20, 25, and 30 min. (**B**,**C**) Sensitivity of conventional heat block and gel electrophoresis of LAMP reaction for the detection of *E. coli* O157:H7 rfbE gene and *S. aureus* (MSSA) spA gene. Amplifications were identified at 30 min for both targets.

Six 10 µL LAMP reaction mixtures with 1 µL, 10^5^ CFU/µL *E. coli* O157:H7 and six 10 µL no target controls (NTC) were prepared as a single experiment set. Identical experiments were also performed with 1 µL, 10^5^ CFU/μL *S. aureus (*MSSA) and NTCs. For each experiment set, the LAMP reaction mixtures were conventionally amplified for 5, 10, 15, 20, 25, and 30 min (Fig. 1A). Once incubated for a set period of time, reaction mixtures were placed on ice, then put through gel electrophoresis. Verified gel of each reaction (0 to 30 min) for target (Fig. 1B) was then completed to determine successful or non-successful amplification. Amplification was detected at 30 min for both targets.

### Contact angle changes during LAMP

**Figure 2:**
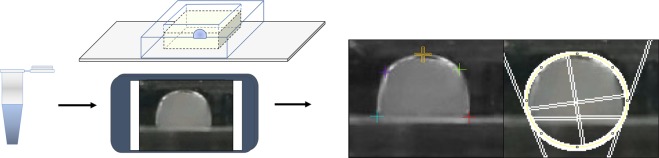
Schematic of real-time monitoring of contact angle during droplet LAMP reaction in a PDMS well (single chamber version), followed by contact angle measurement via ImageJ and Contact Angle plugin. Ellipse-fit was used.

To indirectly measure the interfacial effects of the LAMP reaction, droplets were placed in a PDMS chip filled with mineral oil where contact angle changes were measured, as depicted in Fig. 2. 10 μL LAMP reaction mixtures were prepared with 1 μL of 10^5^ or 0 (NTC) CFU/μL *E. coli* O157:H7 bacteria target or 1 μL of *S. aureus* (MSSA) cross-reactive sample where designated *E. coli* O157:H7 primer set was used. As shown in Supplementary Fig. S2, target bacteria *E. coli* had greater change in contact angle in comparison to the cross-reactive target *S. aureus* (the primers were designed to target *E. coli* O157:H7 but not *S. aureus*) and NTC sample throughout the 30 min reaction.

### Sensitivity analysis in a buffer sample

**Figure 3:**
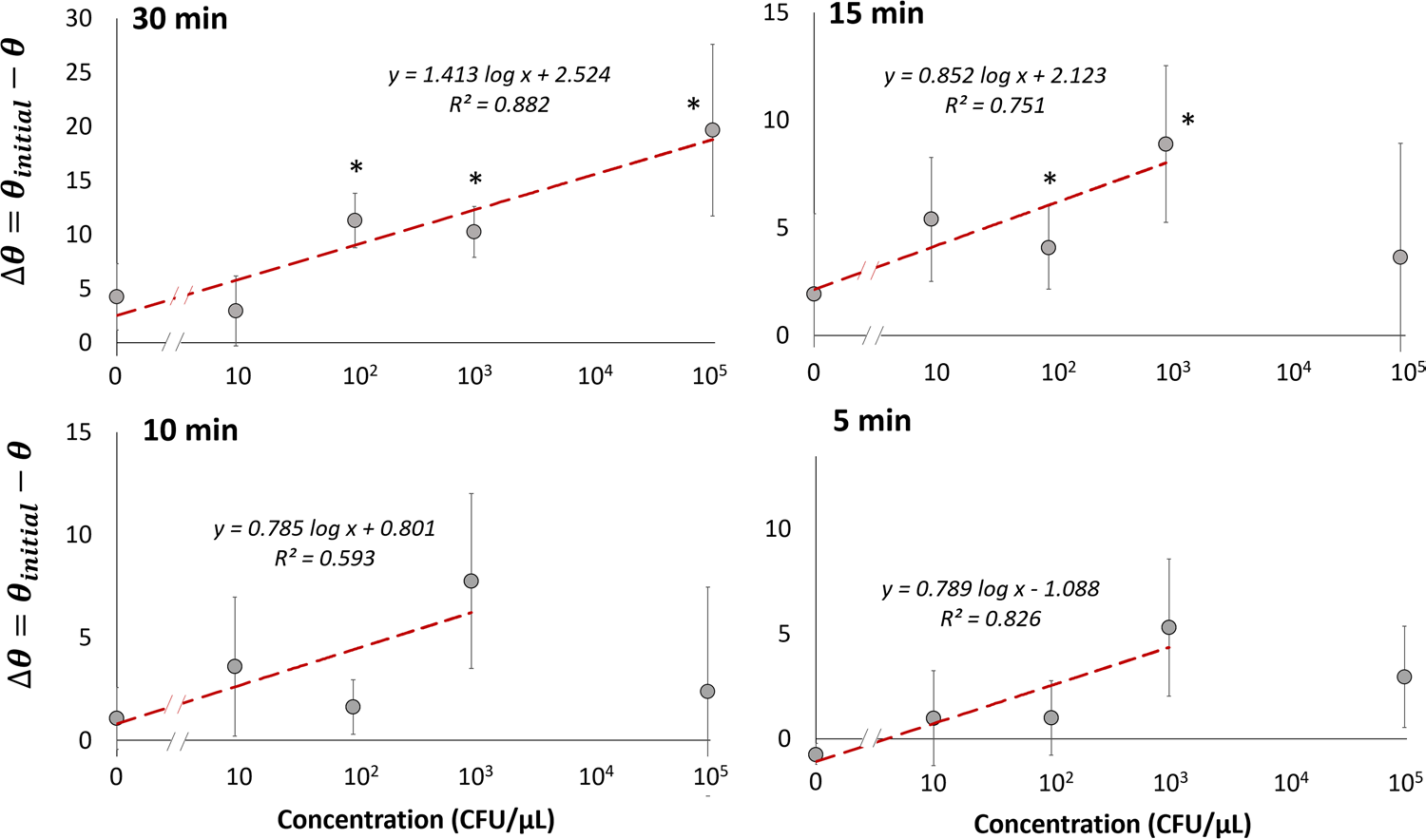
Change in contact angle (Δθ = θ_initial_ − θ) for varying *E. coli* concentrations from 0 to 10^5^ CFU/μL measured at 30 min, 15 min, 10 min, and 5 min. Averages of three different experiments. Error bars represent standard errors. *Indicates substantial changes from the initial contact angles with 95% confidence.

*E. coli* bacteria solutions were serially diluted from 10^5^ to 10 CFU/µL (excluding 10^4^ CFU/µL), which were used as target samples utilizing previously stated LAMP droplet mixtures in a PDMS chamber filled with mineral oil. Images of droplets were captured every 30 s to monitor change in contact angle throughout the 30 min reaction. In Fig. 3 the calculated change in contact angle (Δθ = θ_initial_ − θ) at 30, 15, 10, and 5 min is shown in relation to the concentration of bacteria. Starting at 5 min (Fig. 3C), change in contact angle shows a fairly moderate R^2^ value of 0.826 when fitted to a logarithmic regression. This included bacteria concentrations of 0 (NTC), 10, 10^2^, and 10^3^ CFU/µL ranging from contact angle change of −0.74° to 5.1°. After 10 and 15 min (Fig. 3B,C) excluding 10^5^ CFU/µL concentration, R^2^ values were 0.593 and 0.751 respectively when fitted to a logarithmic regression, displaying inferior proportionality between Δθ and log bacteria concentration. Δθ ranged from 1.07° to 7.75° at 10 min, and from 1.91° to 8.88° at 15 min. At 30 min (Fig. 3A) a fairly good R^2^ value of 0.882 was reported in correspondence to a logarithmic regression, which included all bacteria concentrations from 0 to 10^5^ CFU/µL. Overall, the shortest time-to-result for significant detection was observed for 10^3^ CFU/µL bacteria concentration, with a detection time as soon as the first 5 min of the reaction. At 30 min, statistical difference between target and NTC was seen for all concentrations higher than or equal to 10^2^ CFU/µL, which is the limit of detection (LOD) of this assay.

### Sensitivity analysis in a blood sample

**Figure 4:**
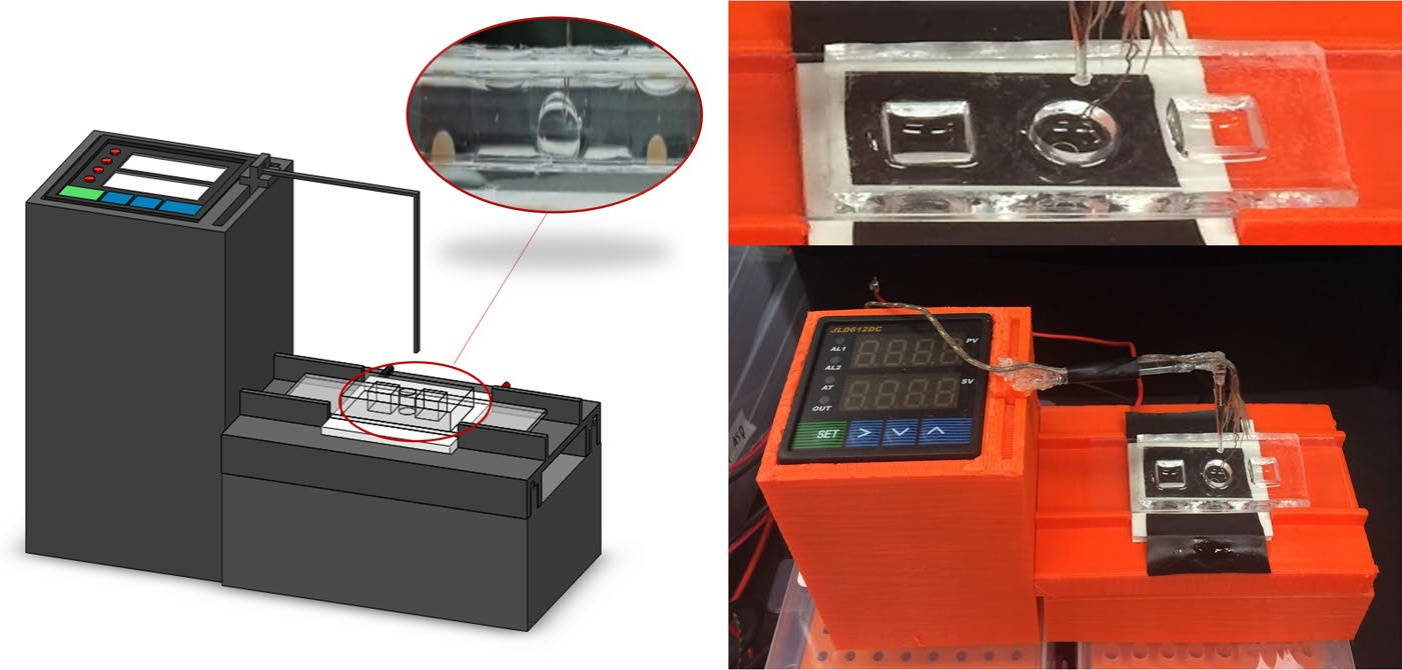
Fully-integrated, all-in-one PID-controlled device for real-time monitoring of contact angle during droplet LAMP reaction in a PDMS-based, two-chamber version chip, for side-by-side comparison of target and NTC droplets.

**Figure 5:**
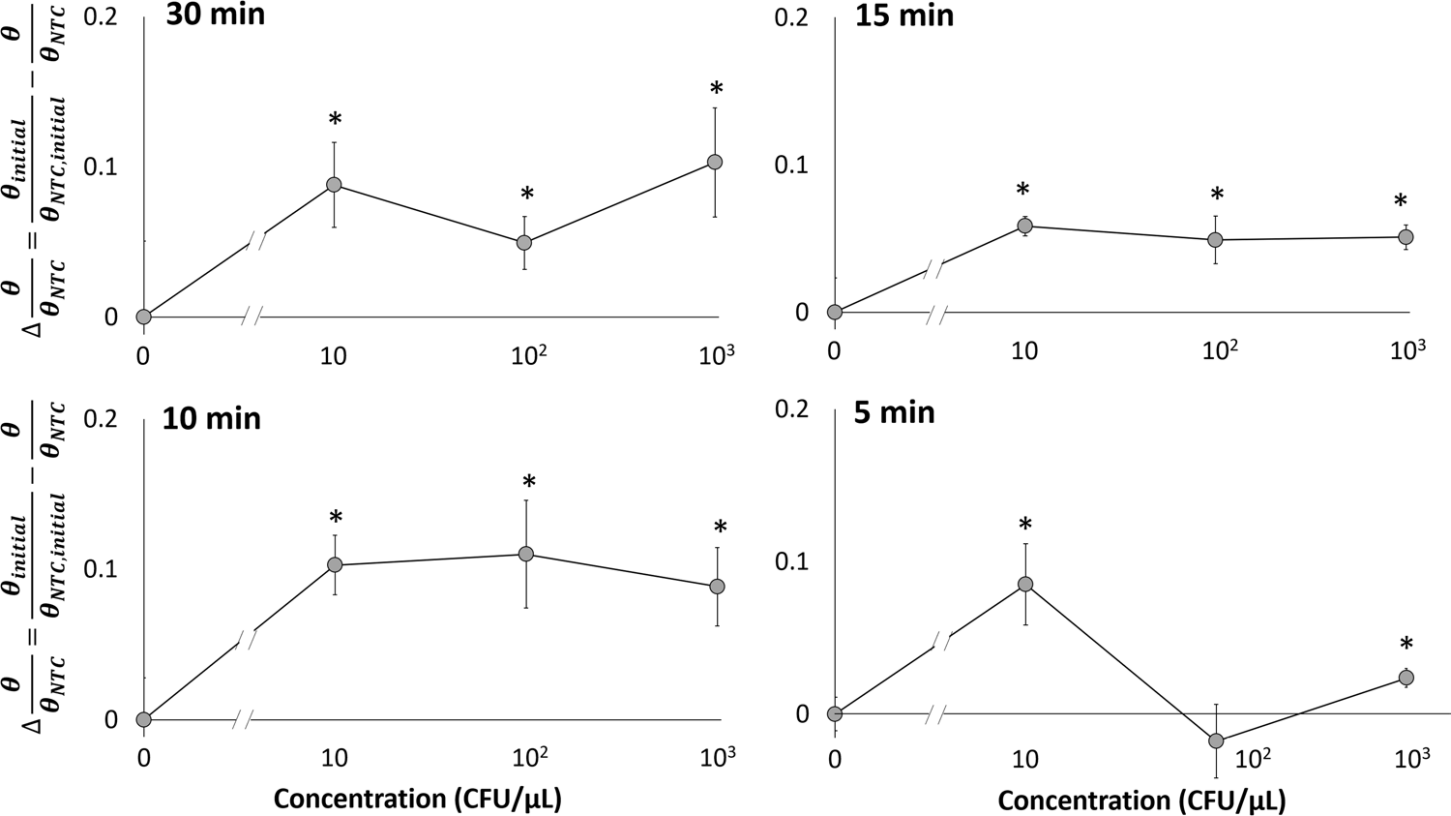
Normalized change in contact angle (Δθ/θ_NTC_ = θ_initial_/θ_NTC,initial_ − θ/θ_NTC_) with varying concentrations from 0 to 10^3^ CFU/μL in 5% whole blood at 30 min, 15 min, 10 min, and 5 min. Averages of three different experiments. Error bars represent standard errors. *Indicates substantial changes from the initial contact angles with 95% confidence.

In this model, *E. coli* bacteria was serially diluted in 5% whole blood from 10^3^ to 10 CFU/µL and used as the 1 µL target volume for the LAMP reaction. As each blood sample was different from each other, the resulting contact angle also varied from sample-to-sample. Therefore, it became necessary to monitor the contact angles of target and NTC simultaneously and normalize the target’s contact angle with that of NTC. To this end, a PID-controlled prototype was designed and assembled to run a contact angle change analysis in a custom-made, two chamber version PDMS chip as shown in Fig. 4. Again, images were taken every 30 s throughout the 30 min LAMP reaction and used to measure contact angle. Prior to calculating change in contact angle, all angles were normalized to those of NTC droplets: Δθ/θ_NTC_ = θ_initial_/θ_NTC,initial_ − θ/θ_NTC_, where positive Δθ/θ_NTC_ correlates to decrease in θ/θ_NTC_. This was done to take into account the interfacial effects caused by the fast oil-water diffusion and adsorption of blood proteins to the oil-water interface. As shown in Supplementary Fig. S3, all bacteria concentration showed a change in θ/θ_NTC_ at each time interval between 5 and 30 min. At 5 min, 10 and 10^3^ CFU/µL show statistical difference compared to NTC with Δθ/θ_NTC_ of 0.085 and 0.023 respectively (Fig. 5D). At 10 min, all bacteria concentrations from 10 to 10^3^ CFU/µL showed statistical difference to NTC with Δθ/θ_NTC_ of 0.10, 0.11, and 0.088 respectively (Fig. 5C). Similar to 10 min, 15 min showed statistical difference compared to NTC for all bacteria concentrations with Δθ/θ_NTC_ ranging from 0.058 to 0.049 (Fig. 5B). At 30 min, Δθ/θ_NTC_ ranged from 0.10 to 0.049 for all bacteria concentrations (Fig. 5A). The overall trends observed showed a binary logistic, where when target bacteria was present, Δθ/θ_NTC_ was observed with significant detection as soon as 5 min for 10 CFU/µL and for all bacteria concentrations at 10 min.

## Discussion

### Advantages of droplet LAMP

The proposed method and device are well-suited for portable and low-cost nucleic acid-based identification of bacterial infection, which is also demonstrated in complex sample matrices. In comparison to other nucleic acid amplification devices^39–41^, the developed platform requires neither moving parts nor liquid flow. Within the droplet LAMP device, samples and thermocouple components are stationary within their respective reservoirs, which reduces the risk for contamination and other factors that can contribute to false assay results. Additionally, the contact angle measurement of a droplet is less susceptible to external forces and vibrations. Interfacial tension has most commonly been measured via pendant droplet analysis, which is quite vulnerable to external vibration and gas entrapments within the needle tip; furthermore, it is difficult to deliver uniform heat to the droplet itself due to the presence of a needle.

The proposed method occurs at an isothermal temperature. Comparatively, PCR-based point-of-care devices require large temperature gradients: 94 °C for denaturation, 72 °C for extension, and 50–65 °C for annealing. Such broad temperature ranges must also be timed precisely to prevent undesirable, non-specific annealing and extension^42,43^, requiring close supervision. As mentioned previously, the droplet LAMP device has an integrated PID controller, automatic image capturing, and analysis system. Such integration allows for minimal supervision during reaction period and fast contact angle analysis, allowing successful identification of target bacterial presence.

### Assay performance

The change in contact angle of LAMP droplets without blood (buffer system) had a logarithmic regression fitted to the bacteria concentration at 30, 15, 10, and 5 min. Relatively good R^2^ values were found at 5 min from 1 to 10^3^ CFU/μL and at 30 min from 1 to 10^5^ CFU/μL. A significant difference was determined in as soon as 5 min for the buffer system with 10^3^ CFU/μL bacteria concentration, which was the limit of detection (LOD) at 5 min. LOD was reduced to 10^2^ CFU/μL at 15 min and at 30 min. The contact angle analysis of LAMP reactions in a blood sample was determined to be a binary assay, i.e., target or no target presence. This was demonstrated in as fast as the first 5 min of reaction for 10 CFU/μL (LOD). A significant difference was identified for all concentrations tested, from 10 to 10^3^ CFU/μL as early as 10 min. Given the small sample volume of 1 μL, the LOD of 10 CFU/μL is close to the lowest possible level of detection, despite the presence of whole blood components. The LODs of many commercial rapid kits for bacterial infection are typically in the range of 10^5^–10^6^ CFU/mL (=10^2^ – 10^3^ CFU/μL), which have frequently been used to identify urinary tract infection (UTI) from urine^44^, gonorrhea from urine^44^, and Streptococcus from throat swab^45^. Typical concentrations of commensal bacteria on human skin can be as high as 10^5^ CFU/cm^2^, and they increase to 10^7^ – 10^8^ CFU/cm^2^ with symptomatic skin and would infections^46^. Overall, the prototype device demonstrated that the presence of bacteria could easily be identified by monitoring the change in contact angle for target and NTC droplets side-by-side, i.e., paired comparison. Compared to other conventional quantitative LAMP assays, our method showed much shorter assay times. Only a relatively small number of amplified molecules are necessary to diffuse towards the oil-water interface to alter the interfacial tension, and subsequently contact angle, thus making it a fast time-to-result assay.

### Molecular diffusion and adsorption of LAMP amplicons to oil-water interface

**Figure 6:**
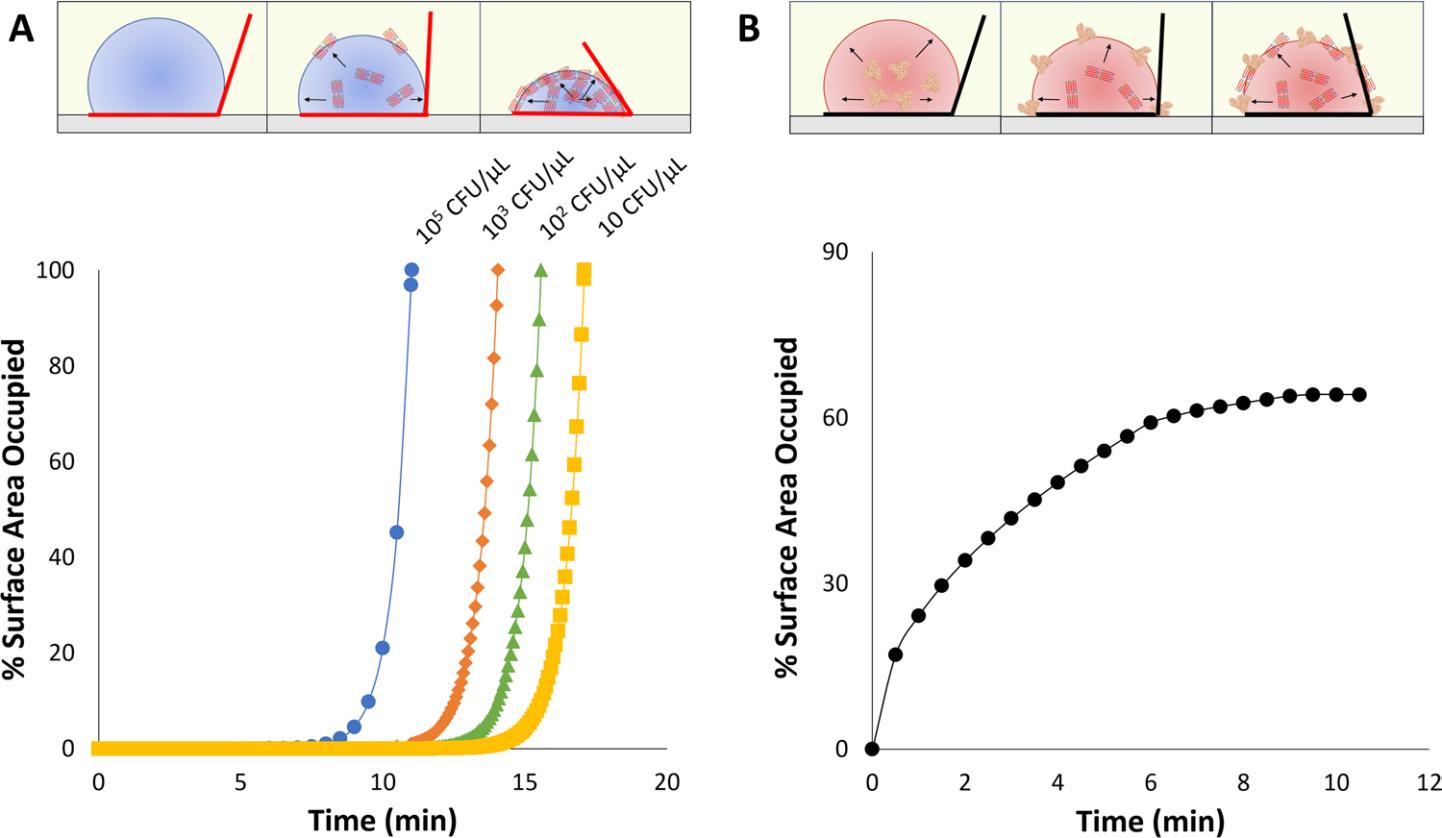
(**A**) Illustration and simulation of % surface area occupied by the diffusion of 965-bp amplicon to the oil-water interface with doubling time of 28.1 s and growth constant k of 0.022 for initial bacteria concentrations from 10^5^ to 10 CFU/µL in buffer system (without blood). (**B**) Illustration and simulation of % surface area occupied by the diffusion of blood protein species (albumin, IgG, and fibrinogen) to the oil-water interface, depicting slower interface occupation by blood proteins than those by amplicons while leaving substantial unoccupied area that can accommodate amplicons.

As illustrated in Fig. 6A, nucleic acid amplification occurs inside of the droplet, followed by the sequential adsorption of produced amplicons to the oil-water interface due to their (loop-shaped) complex geometry, which compromises solvation in water. The movement of the amplicons, especially ones with higher molecular weights, is driven by molecular diffusion. Diffusion constants of nucleic acids have been well studied in relation to their size, due to the wide use of electrophoresis techniques for size separation. Models such as the Nernst/Stokes-Einstein equation, Zimm’s theory, and Kratky-Porod equation have been widely applied^47,48^ to simulate both translational and rotational diffusion of both small rod-like and polymeric chain nucleic acid structures ranging from 30 to 5000 base pairs (bp). Generally, such investigations concluded that the diffusion coefficient and overall velocity are inversely related to bp length. Products of isothermal loop-mediated amplification are large dumbbell-like structures with a wide range of bp lengths. Diffusion of such high molecular weight molecules is difficult and slow, especially when their concentration is very high (molecular crowding).

Both PCR and LAMP amplicons consist of double stranded DNA, which is partially hydrophilic due to its sugar-phosphate backbone^49^. Its interior is more hydrophobic allowing the backbone to be held together by van der Waals forces, leading to overall stability of the structure^50,51^. Therefore, amplicons’ surfaces are less hydrophilic (or become relatively hydrophobic). Such amphiphilic character is more pronounced with LAMP amplicons due to their complex geometry, making them to be excluded in polar solvents and concentrated at the oil-water interface due to the hydrophobic effect. This is also similarly observed for another type of amphiphilic molecule: proteins^52^.

To model amplicon diffusion within a LAMP droplet, a combined exponential growth and Fick’s diffusion-based model was used varying initial *E. coli* bacteria concentration. The model takes into account the exponential production of amplicons and the corresponding diffusivity constant based on the amplicon’s molecular weight. The model assumes a hemi-spherical droplet shape (as the initial contact angle is close to 90° in most cases) to determine the maximum number of available spaces for amplicons at the oil-water interface (calculated by hydrodynamic radii^53,54^). The model varies the initial bacteria target concentration (*C*_0_, CFU/µL) to determine the interface concentration of amplicons (*n*, amplicons per cm^2^) at the given time *t*. An amplicon length of 965 bp was used in the simulation in Fig. 6A, where 965 bp is 5 times the length of target gene, 193 bp (rfbE gene from *E. coli*). Amplicon length of 1930 bp (10 times the target gene length) was also modeled (Supplementary Fig. S4). Exponential growth constant (*k*, s^−1^) and diffusivity constant (*D*, cm^2^ s^−1^) of produced amplicons were chosen based on literature^55,56^ (specific values used can be found in Supplementary Tables S1 and S2). Cumulatively, the model determines *n* with respect to time, *t*:

1 $$n(t)=2{C}_{0}{e}^{kt}\sqrt{\frac{Dt}{\pi }}$$

Similarly, to explain and simulate the diffusion and adsorption of blood proteins (albumin, IgG, and fibrinogen^57^) in a blood sample, a Fick’s diffusion equation was utilized.

2 $$n(t)=2{C}_{0}\sqrt{\frac{Dt}{\pi }}$$

Using the equations (1) and (2), the % fractions of surface area occupied by amplicons and blood proteins were determined using the amplicons’ or blood proteins’ hydrodynamic radii.

As illustrated in Fig. 6A, LAMP droplets (buffer system) with target presence internally produce amplicons, which then diffuse and adsorb to the oil-water interface reducing interfacial tension, thus changing the droplet contact angle (Fig. 3). It was also shown that greater contact angle change was observed with increasing initial bacteria concentration. In comparison, change in droplet contact angle remained relatively constant in the LAMP droplets with no target presence and with cross-reactive target. As shown in Fig. 6B and Supplementary Fig. S4, the slopes of all curves are quite steep, indicating rapid diffusion and adsorption of amplicons to the oil-water interface. The time for amplicon saturation is, therefore, dictated primarily by the production rate of amplicons, which is a function of initial bacteria concentration. This allows the proposed method to quantify the initial bacteria concentration.

With blood present for the blood sample, the trend in contact angle change was binary, i.e., target or no target presence. In this model, both amplicons and blood proteins are competing for the available spaces at the oil-water interface. With NTC (=0 CFU/µL, i.e. no amplicon presence), the surface is occupied only by blood proteins, which is a relatively slow process that is unable to saturate the entire surface – it takes 7 min to reach maximum surface coverage of approximately 60% (Fig. 6B). Proteins are unable to saturate the whole surface due to (1) spreading and flattening at the interface, (2) lateral repulsion between proteins with net positive or net negative charge, especially albumin^55^, and (3) solvation of proteins that may interfere with additional protein adsorption. The remaining 40% of the interface is left for amplicons to arrive. However, there are not enough amplicons produced by this time (7 min) for the range of concentrations tested (10 – 10^5^ CFU/µL) as shown in Fig. 6A and Supplementary Fig. S4. Therefore, amplicon adsorption is occurring after the surface is occupied with blood proteins. This delayed adsorption of the amplicons and competition with the blood proteins is illustrated in Fig. 6B. While the additional amplicon adsorption did result in further contact angle change as shown in Fig. 5, its competition with blood proteins rendered this method unable to quantify initial bacteria concentration in a blood sample. Despite this, the model successfully identified the presence of bacteria in as little as 5 min with as low as 10 CFU/µL initial concentration, which is a significant improvement compared to that in a buffer sample. In addition, the error bars are generally much smaller than those in a buffer sample, presumably due to the presence of blood proteins at the interface, most notably albumin, which can “passivate” the interface.

## Conclusions

A droplet LAMP-based POC platform was designed and tested for detecting bacteria (*E. coli* O157:H7) from diluted (5%) whole blood samples, utilizing smartphone-based contact angle measurements. Monitoring of droplet contact angle was demonstrated in a disposable, two-chamber PDMS chip, incorporating a compact, PID temperature controller and a smartphone.

Analysis of droplet contact angle in a buffer sample showed decrease in contact angle with target presence, with log-linear quantifiable capabilities from 10 to 10^3^ CFU/μL *E. coli* in as little as 5 min and from 10 to 10^5^ CFU/μL by the end of the 30 min reaction. In comparison, NTC droplets (zero bacteria concentration) did not show significant change in contact angle throughout the 30 min isothermal reaction. Analysis of droplet contact angle in a blood sample (bacteria spiked into 5% diluted whole blood) showed, again, decrease in contact angle with target presence. However, no bacteria concentration-dependent relationships were distinguishable, rendering this method binary (i.e. target or no target) in a blood sample. Binary results were determined in as little as 5 min for 10 and 10^3^ CFU/μL *E. coli* and in 10 min for all concentration tested (10 to 10^3^ CFU/μL). Thus, we have demonstrated an assay time of 5–10 min, sufficiently fast for physicians to make clinical decisions, with low limit of detection as low as 10 CFU/μL sample.

Molecular diffusion and interfacial adsorption models were developed for blood proteins as well as exponentially produced amplicons. Due to the higher concentrations of blood proteins and smaller number of amplicons early in the assay, interfacial adsorption is initially dominated by blood proteins, which are later replaced by exponentially producing amplicons that compete with the blood proteins. While the assays with blood samples show only binary assay results, they are much more reproducible with smaller error bars, which is a unique advantage of this method. In other biosensing methods, whole blood generally compromises the assay reproducibility or sensitivity. This proposed method can be further investigated and applied in other complex biological matrices such as urine, saliva, other bodily fluids, feces, and soft tissues for fast (<10 min) nucleic acid amplification.

## Supplementary information

Supplementary Information

## Data Availability

All data generated or analyzed during this study are included in this published article and the supporting information.
