# Supplementary material for: Interfacial Effect-Based Quantification of Droplet Isothermal Nucleic Acid Amplification for Bacterial Infection
Source: Sci Rep. 2019 Jul 3;9:9629. doi: 10.1038/s41598-019-46028-8 (PMC6610113; doi:10.1038/s41598-019-46028-8)
Supplement: Supplementary file 1 — Supplementary Information [file 41598_2019_46028_MOESM1_ESM.pdf]

SUPPLEMENTARY INFORMATION

Interfacial Effect-Based Quantification of Droplet Isothermal Nucleic Acid Amplification for Bacterial Infection

Tiffany-Heather Ulep, Alexander S. Day, Katelyn Sosnowski, Alexa Shumaker and Jeong-Yeol Yoon

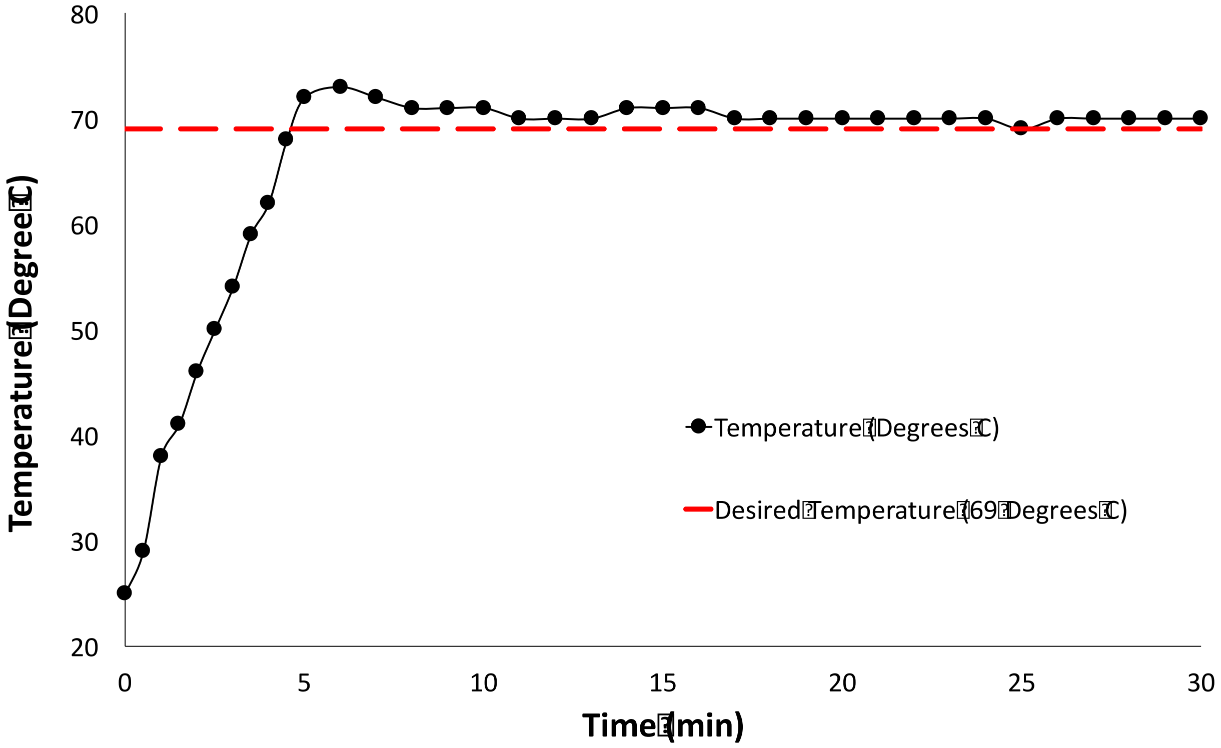

Supplementary Figure S1. PID Temperature controller temperature characterization with tuned parameters to ensure constant 69°C throughout 30 min LAMP reaction (P = 0.1%, I = 12 sec, and D = 28 sec).

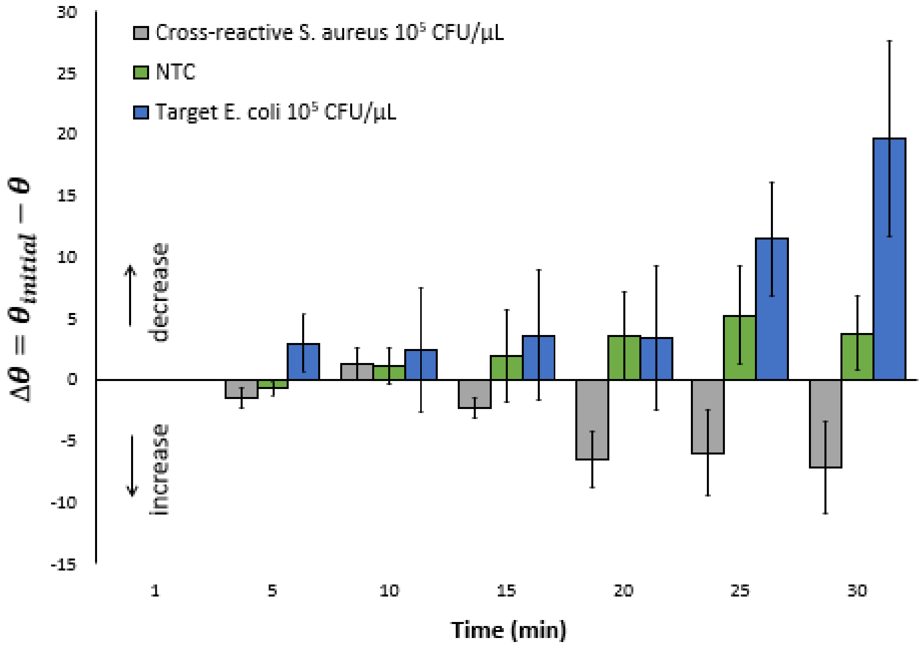

Supplementary Figure S2. Change in contact angle ($\Delta\theta = \theta_{initial} - \theta$) of droplet LAMP targeting *E. coli* O157:H7 10^5 CFU/µL, cross-reactive sample of *S. aureus* 10^5 CFU/µL, and NTC.

Target 10^5 CFU/µL *E. coli* contact angle measurements showed continued decrease over time with Δθ (= $\theta_{initial} - \theta$) of 3.0° as soon as 5 min and a maximum Δθ of 21° at 30 min. Meanwhile, the cross-reactive *S. aureus* had an increase in contact angle, with Δθ of –1.5° at 5 min and a maximum Δθ of –7.4°. NTC's Δθ was –0.74° at 5 min and a final Δθ of 3.7° at the end of the 30 min reaction. Significant difference was identified with *S. aureus* cross-reactive target at >20 min amplification. Therefore, overall change in contact angle is solely attributed to amplicon production and potentially its adsorption to the interface.

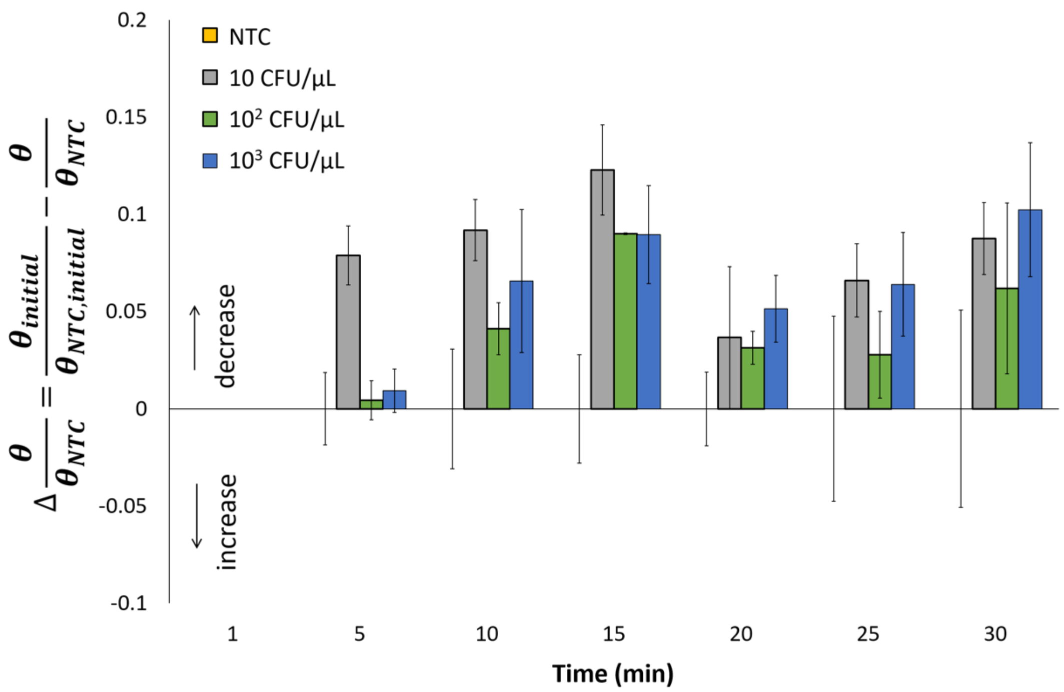

Supplementary Figure S3. Normalized change in contact angle ($\Delta\theta/\theta_{NTC}$) for varying concentrations from 0 to 10^3 CFU/µL throughout 30 min LAMP reaction in 5% whole blood. Averages of three different experiments. Error bars represent standard errors.

Supplementary Table S1. Calculation of molecular weight, radius, and area for 193-, 965-, and 1930-bp amplicons to evaluate the % surface area occupied. Molecular weight = n x 607.4 + 157.9[59].

Base pair length (bp)	n (number of nucleotides)	Molecular weight[59] (g/mol)	Radius[60] (nm)	Area (cm^2)
193	386	234,614	4.07	5.22×10^{-13}
965	1930	1,172,440	6.96	1.52×10^{-12}
1930	3860	2,344,722	8.77	2.42×10^{-12}

Supplementary Table S2. Diffusion constants for 193-, 965-, and 1930-bp amplicons, as well as albumin, IgG, and fibrinogen to evaluate the % surface area occupied.

Molecule	D, diffusion constant ($cm^2\ s^{-1}$)	References
193-bp amplicon	1.11×10^{-7}	61, 62
965-bp amplicon	3.48×10^{-8}	
1930-bp amplicon	2.11×10^{-8}	
Albumin	6.1×10^{-7}	63
IgG	4.0×10^{-7}	
Fibrinogen	2.0×10^{-7}	

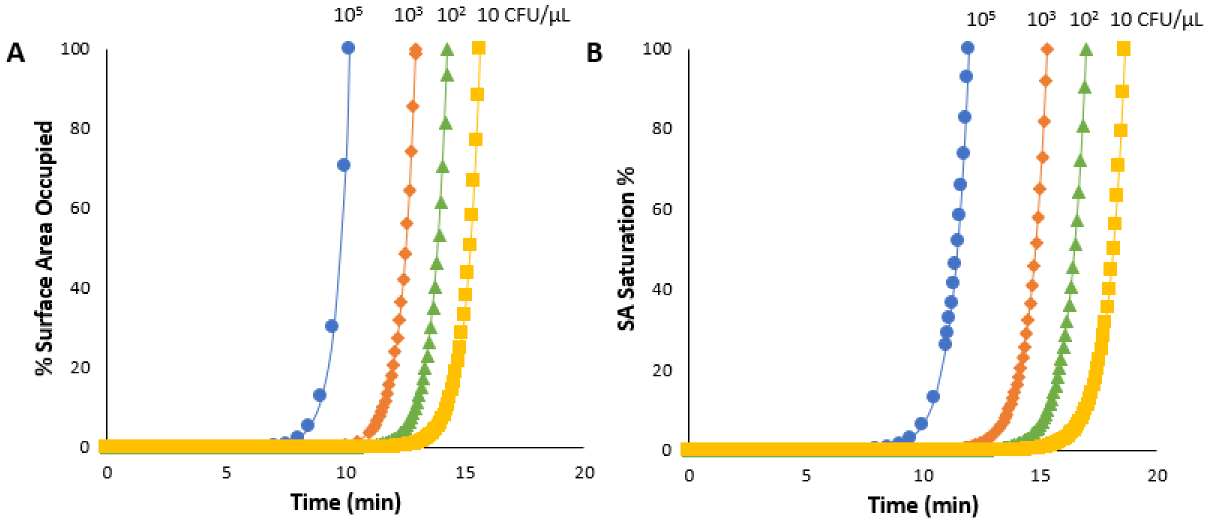

Supplementary Figure S4. Simulation of % surface area occupied by the diffusion of 193-bp amplicon (A) and 1930-bp amplicon (B), in comparison to that of 965-bp amplicon shown in Figure 6A. Doubling time is 25.2 s for 193-bp and 31 s for 1930-bp, and growth constant k is 0.0275 for 193-bp and 0.0223 for 1930-bp.
